# Trends in Physician Emigration from Conflict-Affected Countries, 2006-2021

**DOI:** 10.1007/s11606-025-09791-1

**Published:** 2025-08-05

**Authors:** Tarun Ramesh, Gregory D. Wozniak, Marcela Horvitz-Lennon, Fang Zhang, Hao Yu

**Affiliations:** 1 Department of Population Medicine, Harvard Medical School and Harvard Pilgrim Health Care Institute, Landmark Center, 401 Park Drive, Suite 401 East, Boston, MA 02215 USA; 2 American Medical Association, 330 N. Wabash Ave., Suite 39300, Chicago, IL 60611 USA; 3 RAND Corporation, 20 Park Plaza, 9th Floor, Suite 910, Boston, MA 02116 USA

Conflict-affected countries (CACs) typically experience worse health outcomes and an increased need for healthcare services, yet conflict has been identified as a ‘push’ factor for physician emigration from low- and middle-income countries to high-income countries.^1,2^ However, little is known about the contribution of recent conflicts to this global medical ‘brain drain.^3^’ This study aimed to fill the gap by analyzing CAC physician emigration trends.

## Methods

This retrospective repeated cross-sectional analysis used publicly available data from the Organization for Economic Co-operation and Development (OECD), whose membership comprises of high-income countries. The data identified the origin countries of physicians immigrating to OECD countries from 2006 to 2021.^4^ We used the World Bank’s Classification of Fragile and Conflict-Affected Situations to designate 55 countries that ever experienced any conflicts between 2006 and 2021.^5^

We described trends in the annual number of physicians emigrating from CACs, identified the CACs with the highest number of physician emigrants, and compared physician density per 1,000 by countries’ conflict status. We examined trends in physician emigration over time by plotting the annual number of physician emigrants relative to the year of conflict onset (year 0) in a subsample of 14 CACs observed from 4 years before to 4 years after conflict onset. The study followed STROBE reporting guidelines and was deemed not human subjects research by the corresponding author’s institutional review board.

## Results

**Figure 1:**
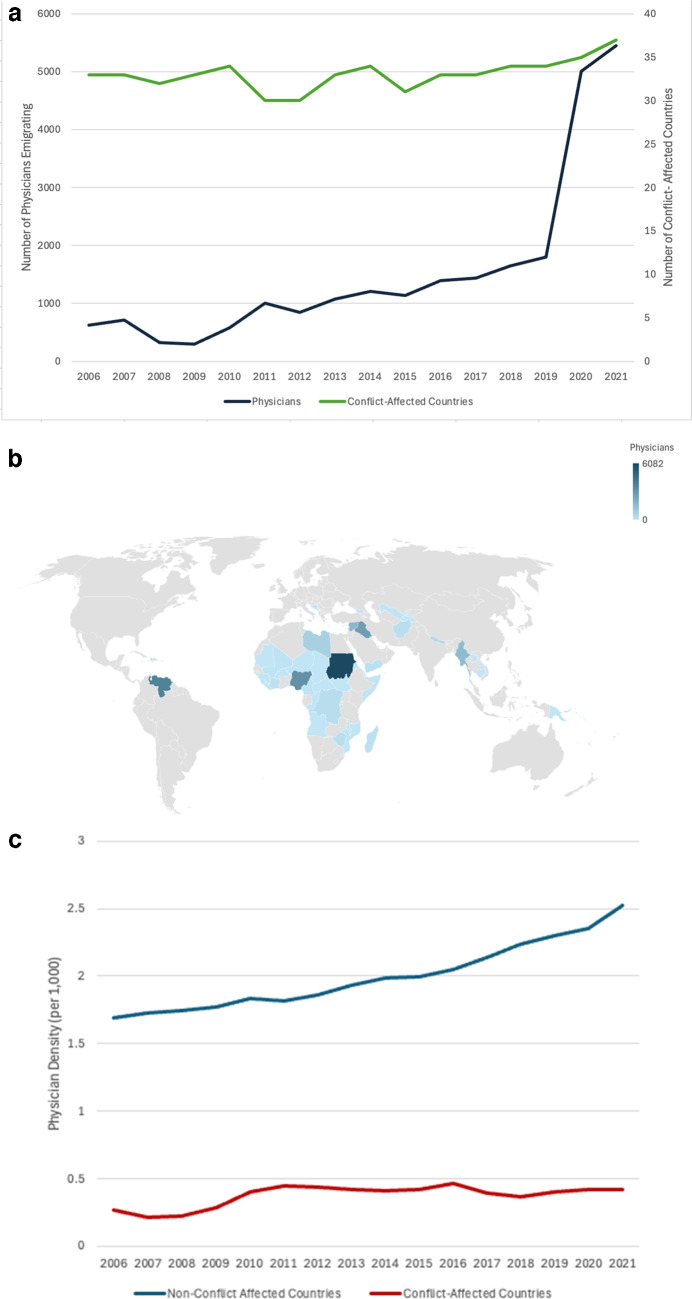
**a** Trends in the Number of Conflict-Affected Countries and Their Physician Emigrants, 2006-2021. A Description: The number of physicians was plotted on the primary y axis labeled as the number of physicians emigrating, while the number of conflict-affected countries was plotted on the secondary y axis labeled as number of conflict-affected countries. There was a total of 55 countries that ever received a conflict designation by the World Bank during our study period. Countries were able to gain or lose the designation over time depending on the World Bank’s methodology, in part based on if they met a threshold of conflict-related mortality. **b** Origin Countries of Physicians Emigrating from the Conflict-Affected Countries, 2006-2021. B Description: The number of physician emigrants from conflict-affected countries during the study period from 2006-2021 was mapped with darker blue representing a greater number of physician outflow from the conflict-affected country. **c** Trends in the Physician Density by Countries’ Conflict Status, 2006-2021. C Description: The mean physician density (per 1,000) was plotted by year for conflict-affected countries and all other countries (i.e. non-conflict affected countries) as classified in the data from the World Bank.

From 2006 to 2021, 24,534 CAC physicians emigrated to OECD countries, with the annual number rising by 772%, from 625 in 2006 to 5,454 in 2021 (Fig. 1A), while the number of CACs rose only slightly (from 33 to 37). Among CACs, the countries with the highest physician emigration included Sudan (6,082), Venezuela (3,847), Nigeria (3,240), Iraq (2,801), and Myanmar (1,672) (Fig. 1B).

**Figure 2:**
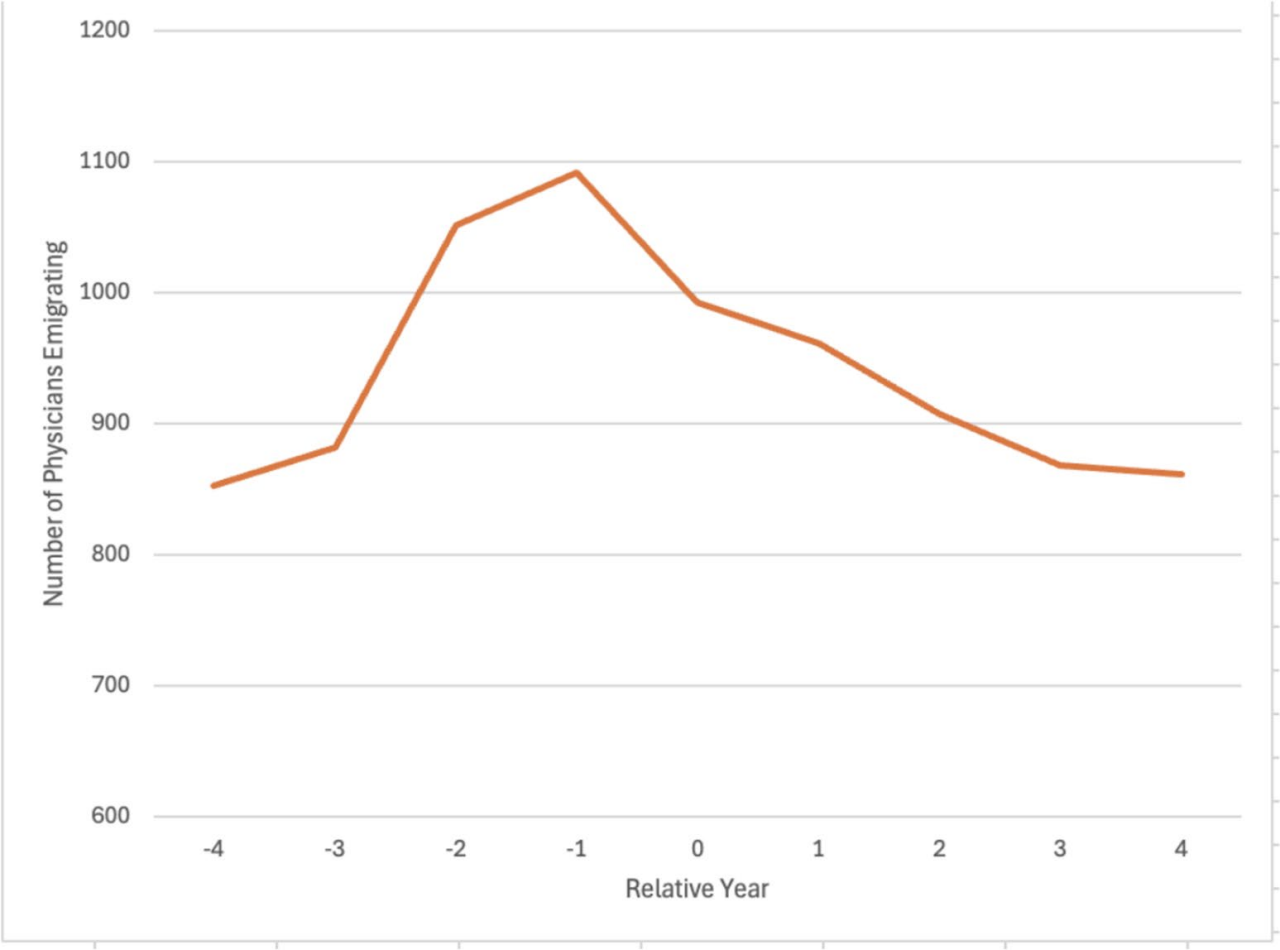
Physician Emigrants from the Conflict-Affected Countries Relative to the Year of Conflict Start (Year =0). Description: We subset our sample to 14 countries that had data for at least 8 years including 4 years prior and 4 years after their conflict. The physician emigration from that country was plotted relative to year 0, which was when they were designated by the World Bank as a conflict-affected country. List of Conflict-affected Countries that had complete data from −4 to +4 year relative to conflict onset: Bosnia and Herzegovina, Georgia, Iraq, Lebanon, Libya, Madagascar, Malawi, Mali, Marshall Islands, Micronesia, Nepal, South Sudan, Syria, and Tuvalu

Compared with non-CACs, CAC physician density remained low (less than 0.5 physicians per 1,000) throughout the study period, with the gap between the two country groups increasing over time; by 2021, physician density was nearly sixfold higher in non-CACs than in CACs (2.51 vs 0.42 per 1,000) (Fig. 1C). The temporal distribution of CAC physician emigrants was roughly bell-shaped, peaking in the year before conflict (Fig. 2).

## Discussion

Physician emigration from CACs has increased more than sevenfold between 2006 and 2021, totaling nearly 25,000 physicians globally. The low physician density in CACs, lower than that of non-CACs and increasingly so over time, highlights the contribution of CAC physician emigration to the substantial physician shortages in CACs, which affect their capacity to address unmet healthcare needs. Physician emigration peaked in the year before conflict initiation, suggesting an anticipatory effect of the conflict on emigration. After the conflict onset, the number of physicians emigrating decreased, which could be due to transportation problems, less medical graduates, inaccurate reporting, or difficulty obtaining a visa for emigration. Overall, our study suggests that policies intended to improve physician supply once a conflict has begun may be less effective because a substantial portion of physician emigration has already occurred. Instead, conflict relief efforts should aim to fill the void and prevent further physician emigration. The 2010 World Health Organization Global Code of Practice for the International Recruitment of Health Personnel intended to reduce global medical ‘brain drain’ by supporting shortage countries. Our findings suggest that the Code has been rather ineffective at stemming CAC ‘brain drain.’ Therefore, international policies targeting CAC physician emigration are urgently needed given the ongoing conflicts across the world.^6^

Study limitations include that only OECD countries were included as destination countries and that we focused on physician migration rather than other types of healthcare professionals. Future studies should examine how conflicts affect the migration of nurses and other healthcare professionals and the effectiveness of policies to promote the return of physicians and other healthcare workers to their countries of origin.

## Data Availability

Data was publicly available.
